# Functional Magnetic Resonance Connectivity in Patients With Temporomadibular Joint Disorders

**DOI:** 10.3389/fneur.2021.629211

**Published:** 2021-04-12

**Authors:** Felice Festa, Chiara Rotelli, Antonio Scarano, Riccardo Navarra, Massimo Caulo, Monica Macrì

**Affiliations:** ^1^Department of Medical, Oral, and Biotechnological Sciences, University of G. d'Annunzio Chieti-Pescara, Chieti, Italy; ^2^Department of Medical, Oral, and Biotechnological Sciences and CeSi Met, University of G. d'Annunzio Chieti-Pescara, Chieti, Italy; ^3^Department of Clinical Sciences and Bio-imaging, University of G. d'Annunzio Chieti-Pescara, Chieti, Italy

**Keywords:** TMD, TMJ, teeth clenching, trigger points, fMRI, facial pain management, myofascial pain, headache

## Abstract

Myofascial pain in the masticatory region, generally referred to as headache, is a common temporomandibular disorder (TMD) characterized by the hypersensitive regions of the contracted skeletal muscle fibers. A correct clinical treatment of myofascial pain has the potential to modify the functional activation of cerebral networks associated with pain and unconscious teeth clenching, specifically the pain network (PN) and default mode network (DMN). In this study, research is presented as a case series of five patients with myofascial pain: three were diagnosed with intra- and extra-articular disorders, and two were diagnosed with only extra-articular disorders. All five patients received gnathological therapy consisting of passive splints and biofeedback exercises for tongue–palatal vault coordination. Before and after treatment, patients underwent pain assessments (through measures of visual analog scales and muscular palpation tests), nuclear magnetic resonance of the temporomandibular joint, and functional nuclear magnetic resonance of the brain. In each patient, temporomandibular joint nuclear magnetic resonance results were similar before and after the gnathological treatment. However, the treatment resulted in a considerable reduction in pain for all patients, according to the visual analog scales and the palpation test. Furthermore, functional nuclear magnetic resonance of the brain clearly showed a homogeneous modification in cerebral networks associated with pain (i.e., PN and DMN), in all patients. In conclusion, gnathological therapy consisting of passive aligners and biofeedback exercises improved myofascial pain in all five patients. Most importantly, this study showed that all five patients had a homogeneous functional modification of pain and default mode networks. Using passive splints in combination with jaw exercises may be an effective treatment option for patients with TMD. This research could be a starting point for future investigations and for clinicians who want to approach similar situations.

## Introduction

Temporomandibular disorder (TMD) is a general term used for several clinical issues involving the masticatory muscles and the temporomandibular joint (TMJ), and is one of the most common pathologies of the maxillofacial region in patients between 20 and 40 years. About 33% of the population has at least one symptom of TMD, and between 3.6 and 7.0% require prompt treatment. Furthermore, the concomitant incidence of anxiety and stress in patients exacerbate the TMD symptoms. TMD etiology is related to chronic pain, teeth grinding, and cervical spine problems. Signs and symptoms of TMD include pain, malocclusion, TMJ dysfunction, joint noises, deviation in opening or closing, and restricted jaw movement. Therefore, as an antalgic mechanism, the patient limits their own mandibular movements (1).

Unconscious teeth clenching constitutes a persistent microtrauma of muscles and articulation and is one of the main etiological factors of myofascial pain. It is characterized by regions of contracted motor fibers, defined as “trigger points,” which are the source of constant deep pain and can cause central excitatory effects, commonly referred by patients as headache. The diagnosis of myofascial pain is easily made through medical history and physical examination. The diagnostic suspicion is based on a history of chronic daily headaches and facial pain without evidence of neurological or intracranial abnormalities.

The goals of TMD treatment are to alleviate and/or reduce pain to improve mandibular function. Several procedures have been used as treatment methods, such as drug therapies, surgical and non-surgical procedures, dental appliances, physical therapy, and behavioral and psychosocial interventions. The guidelines of the Royal College of Dental Surgeons of Ontario recommend applying irreversible procedures (e.g., surgical interventions) only after the failure of conservative treatments, if the symptoms are severe and persistent (1).

Functional magnetic resonance imaging (fMRI), by taking the endogenous oxyhemoglobin as a contrast mean, allows inferring functional information on the metabolic activity of different cerebral regions (cerebral networks). Several fMRI studies have shown a permanent modification of pain and behavioral-associated cerebral networks following jaw-related therapeutic interventions (2, 3).

fMRI assesses neural functional activity by measuring the level of intra-tissutal oxyhemoglobin and deoxyhemoglobin, indices of regional blood supply and metabolic activity. The blood oxygenation level-dependent (BOLD) signal can be measured during the execution of a task (task-evoked fMRI) or during rest as a measure of brain functional connectivity (fcMRI). In our setting, we evaluated the fcMRI of two networks, the pain network (PN), and the default mode network (DMN). The PN is the cortical network of the physiology of pain, and the DMN is the network used in the processing of the unconscious processes. DMN is also involved in pain perception. These two cortical networks were analyzed in terms of functional connectivity. This study investigated the neural network activity at rest, in the absence of stimuli. Functional network connectivity is defined as the temporal correlation at rest in cortical networks. The resting functional connectivity represents 80% of the oxygen consumed by brain activities (4).

In this research study, a case series of five patients diagnosed with myofascial pain and treated with passive aligners and biofeedback exercises to teach the patient not to clench their teeth is presented. All the patients underwent quantitative pain assessments and fMRI before and after the gnathological treatment.

## Materials and Methods

The study was performed in the Oral Sciences Department of the University of Chieti G. D'Annunzio. Ethics approval (number 23) was obtained by the hospital's Independent Ethics Committee of Chieti. The study protocol was drawn in accordance with the European Union Good Practice Rules and with the Helsinki Declaration. The sample consisted of a group of five patients who were treated at the Orthodontics and Orofacial Pain Department for TMD disorders. Each patient signed an informed consent form before the study.

The study lasted 1 year: 6 months were used to recruit the patients, 3 months for follow-up of the recruited subjects, and 3 months for data processing.

### Inclusion Criteria

1) At least 18 years of age.
2) Diagnosis of chronic myofascial pain syndrome of the masticatory muscles.
3) Pain in the jaw muscles at least four times a week and for at least 12 weeks.
4) Average pain severity of 4 on a 10-point scale for at least 1 h per day.
5) Pain in the jaw, temples, face, pre-auricular area, or in the ear during rest or function.
6) Diagnosis of TMDs using MR imaging of the TMJ to evaluate the articular disk, or meniscus, in terms of its morphologic features and its location related to the condyle in both closed- and open-mouth positions.

### Exclusion Criteria

1) Pregnancy.
2) Current opioid use.
3) Claustrophobia.
4) Moderate or severe psychiatric disorder or current use of psychiatric medications.
5) Presence of fibromyalgia or other chronic pain disorder.
6) Diagnosis of metabolic disease, coagulopathy, neurological disorder, vascular disease, or neoplasia.
7) Family history of arthritis or gout.

Also, participants taking non-steroidal anti-inflammatory drugs or paracetamol (acetaminophen) stopped those medications at least 1 day prior to their study appointment.

### Measurements

#### VAS

The pain intensity ratio was estimated by using a visual analog scale (VAS), which consisted of a graphic representation of the patient's face. The patient had to highlight painful areas, specifying the intensity (quantifying it with a value from 0 = No Pain to 10 = Maximum Pain) and frequency of the disturbance, and how it affected everyday life (5).

#### Palpation

Palpation of the temporal, masseter, sternocleidomastoid, digastric, and pterygoid muscles and TMJ was made bilaterally with constant pressure. It consisted of searching for trigger points in the masticatory muscles. Accordingly, these trigger points, once stimulated, tend to produce and provoke headaches through central excitatory effects.

The sensations of pain were classified on a scale from 0 to 3:

- 0: the absence of pain;
- 1: mild pain or apparent discomfort with muscle contraction;
- 2: moderate pain or discomfort with muscle contraction;
- 3: severe pain; the patient “draws back” or “drops in tears” (6).

#### MRI of the TMJ

MRI evaluated the integrity of the temporomandibular joint, disc dislocations, and condyle positions to diagnose intra-articular or extra-articular disorders and to assess changes in the condyle–disc relationship associated with clinical treatment. Each patient underwent TMJ MRI procedures with open-mouth and closed-mouth postures, before and after the treatment.

#### fMRI of the Brain

fMRI of the brain analyzed the functional resting connectivity of the pain network (PN) and default mode network (DMN). The PN represents the cortical network of the physiology of pain, whereas the DMN is the system that processes the unconscious mechanisms involved in pain perception.

The DMN areas that were studied:

- Right occipital lobe (DMN-RIGHT-OCC);
- Left occipital lobe (DMN-LEFT-OCC);
- Right temporal lobe (DMN-RIGHT-TEMP);
- Left temporal lobe (DMN-LEFT-TEMP);
- Posterior cingulate cortex (DMN-PCC);
- Precuneus (DMN-PRECUNEUS);
- Medial pre-frontal cortex (DMN-MPFC).

The PN areas that were studied:

- Anterior cingulate cortex (PAIN-ACC);
- Right insula (PAIN-RIGHT-INSULA);
- Left insula (PAIN-LEFT-INSULA);
- Right somatosensory cortex 1 (PAIN-RIGHT-S1);
- Left somatosensory cortex (PAIN-LEFT-S1);
- Right somatosensory cortex 2 (PAIN-RIGHT-S2);
- Left somatosensory cortex 2 (PAIN-LEFT-S2).

The fMRI of the brain allowed assessment of the *average functional connectivity* concerning PN and DMN networks from the *different functional connectivity matrices*, obtained from the difference between the connectivity matrix at T2 (posttreatment) and the connectivity matrix at T1 (baseline), for each subject. In the matrices, each node corresponds to the numerical value of the interaction of two specific ROIs (regions of interest): the ROI of the row with the ROI of the column. In the difference matrix of each patient, the algebraic sum of all the nodes in each network was calculated. A positive value of average functional connectivity corresponds to a greater functional connectivity at rest in that network after treatment. A negative value corresponds to a lower functional connectivity at rest after treatment.

### MR Data Acquisition and Processing

The MRI data were collected using a GE Medical Systems 3.0 Tesla system with an eight-channel brain-receiving coil.

The protocol used a fast 3D-SPGR sequence with the following parameters:

- TR = 6.9 ms,
- TE = 1.6 ms,
- TI = 450 ms,
- flip angle = 15°,
- matrix = 256 × 256,
- field of view = 25.6 × 25.6 cm,
- 156 axial slices with 1 mm thickness, yielding a voxel size of 1 × 1 × 1 mm.

The scanning parameters provided complete coverage of the brain, midbrain, pons, and cerebellum regions.

CNS abnormalities associated with M-TMD were assessed using several MRI tools.

The first step was the brain extraction and parcellation by using FreeSurfer (7). Then, the 1000 Functional Connectomes Project was followed to obtain residuals from the BOLD images (8). Thereafter, the residuals were registered to MNI template (9), and the FSL toolbox (10) was used to extract the time-course from the selected ROI (11). The functional connectivity matrices and treatment timepoint (T2–T1) differences were calculated using a Python in-house script (12).

### Treatment Protocol

**Figure 1:**
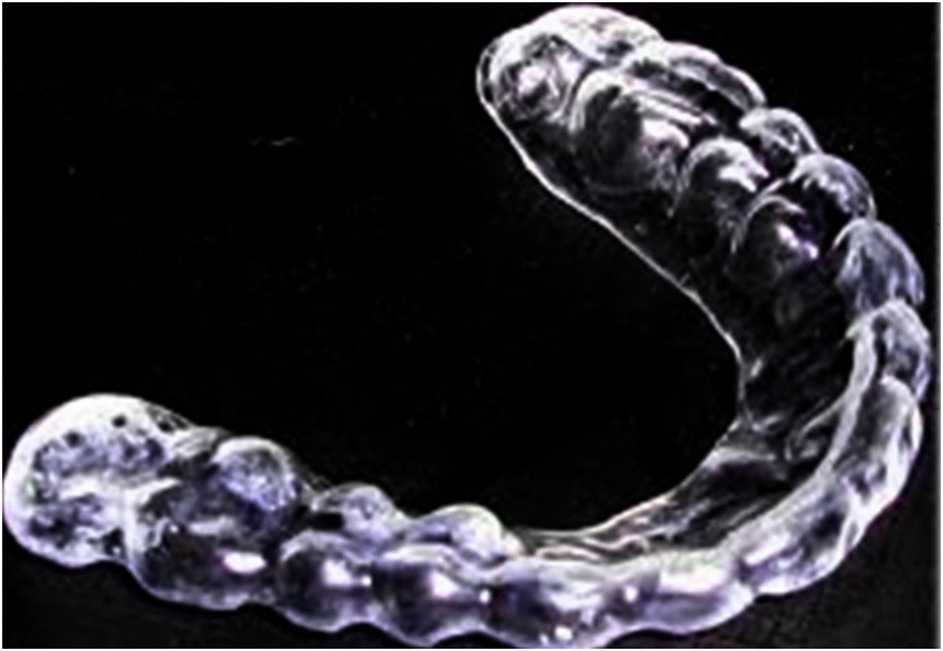
Passive splints made of hard polycarbonate with thickness not exceeding 0.7 mm.

Each patient received two passive splints made of hard polycarbonate that covers all the teeth without pre-established mandibular positions (13) (Figure 1). There was a lower passive aligner splint (LPAS) and an upper passive aligner splint (UPAS). The PAS was made of polycarbonate and was adjusted intraorally, as described by Sears, to avoid the impact of soft tissues. The LPAS was used during the daytime and the UPAS during the night.

While wearing the LPAS, patients performed a biofeedback exercise for 2 min, three times a day (prior to breakfast, lunch, and dinner), with a minimum of 3 h between each exercise, 7 days a week. Biofeedback exercises of the tongue serve to enhance patient awareness of the palatal arches' spatial positioning associated with jaw clenching so that patients can learn to stop or refrain from doing this maladaptive behavior.

During the exercise, patients assumed an upright position or reclined on a hard, flat surface, and were required to follow the accorded three steps:

1. In the first phase, the patient clenched their teeth to fully contract the masseter bilaterally. A light touch with the forefinger on the contracted masseter was applied during maximum contraction. The patient visualized the muscle's volume in a mirror as a swollen tennis ball for 5 s.
2. In the second phase, the patient clenched their teeth to partially contract (~50%) the masseter bilaterally; a light touch with the forefinger was applied during the contraction force, which is about halfway. The patient visualized the muscle's volume in a mirror as a semi-deflated tennis ball for 5 s.
3. In the third phase, the patient was instructed to fully relax their jaw by opening it ~1 mm and applying a light touch with the forefinger on the utterly relaxed masseter. The patient visualized the muscle's volume in a mirror as a completely deflated tennis ball for 5 s.
4. In the fourth phase, the patient touched the tip of the tongue on the top of the palatine vault, approximately between the palatine wrinkles and the flat palate for 5 s.

Then, the patient removed the LPAS for breakfast.

The same exercise was repeated before lunch and before dinner with the LPAS inserted. Biofeedback was timed to occur immediately prior to meals because masseter activation during meals typically causes pain levels to worsen.

The treatment lasted ~3 months. In the 6 months follow-up, a new assessment was made using the VAS and palpation test of temporal, masseter, sternocleidomastoid, digastric, pterygoid muscles, and TMJ MRI, and fMRI of the brain was repeated to evaluate the treatment effect.

Throughout the entire study duration, every patient continued to record in their diaries the extent and intensity of their pain during headaches and treatment sessions/compliance.

### Study Protocol

The study included five patients, three with intra-articular and extra-articular disorders and two with only extra-articular disorders, who were diagnosed by magnetic resonance imaging of the TMJ. All patients were treated using gnathological therapy consisting of passive aligners and biofeedback exercises. The study's patient selection was founded on the diagnosis of TMD based on a standardized and complete clinical examination that fulfills the Research Diagnostic Criteria (RDC TMDs) (14).

In the first phase of the study, all the patients underwent the palpation test and VAS to diagnose myofascial pain disorders. After recruitment in the study, the patients underwent TMJ MRI to assess the TMJ condition and fMRI of the brain to assess functional response.

During the second phase of the study, lasting 6 months, all five patients were treated by gnathological therapy consisting of passive aligners and biofeedback exercises for 2 min, three times a day (prior to breakfast, lunch, and dinner), with a minimum of 3 h between each exercise, 7 days a week.

Patients underwent follow-up appointments once a month, in which the VAS and the palpation test were repeated.

After 3 months, all patients underwent a second TMJ MRI and fMRI of the brain.

All the clinical examinations, splint fitting, and follow-up appointments were performed by the same examiner.

### Statistical Analysis

Paired Student t-tests (pre- and posttreatment, T2-T1) were used to understand the impact of the treatment protocol on VAS scores and PN and DMN average connectivity. The significance threshold for all tests was set at 0.05.

## Results

All five patients exhibited forms of tension headaches but no sort of migraines, diagnosed according to the criteria of the International Classification of Headache Disorders 3rd edition (ICHD-III beta) (15).

In Table 1, the impact of gnathological treatment on pain is reported (VAS and painful areas). Most importantly, by comparing the baseline values (T1) with posttreatment values (T2), the pain symptomatology decreased both in terms of intensity and number of painful areas for each patient. Furthermore, the patients sufficiently complied with the program to constitute a treatment effect.

**Table 1:** Clinical and demographical characteristics of the patients and the impact of the gnathological treatment.

	**Age**	**Gender**	**Kind of TMD**	**VAS at T1**	**VAS at T2**	**Painful areas at T1**	**Painful areas at T2**	**Duration of symptoms**	**Evolution of symptoms**
PT1 (CS)	41	Female	Intra-articular	8	4	Neck, under eyes, shoulders, TMJ, mandible	Neck, shoulders	About 2 years	The symptoms worsened during this period
PT 2 (SS)	22	Female	Intra-articular	8	1	TMJ, around eyes, trapezoids	TMJ	About 1 year	The symptoms worsened during this period
PT 3 (RF)	26	Male	Extra-articular	5	1	Mandible, neck, lumbar area, head	Neck	About 2–3 years	Symptomatology remained constant during this period
PT 4 (AN)	41	Female	Intra-articular	7–8	4	Sinusitis-like symptoms, TMJ, neck, shoulders, pelvis	TMJ	About 15 years	The symptoms worsened during this period
PT 5 (CT)	55	Female	Extra-articular	6	0	Masseter, mandible, maxilla		About 5 years	The symptoms worsened during this period

The improvements were independent of age and gender but dependent on symptom intensity and chronicity at T1. Patient 1, patient 2, and patient 4 had intra-articular and extra-articular disorder; patient 3 and patient 5 had only extra articular disorder. Patient 1 exhibited, after treatment only, residual symptomatology (VAS 4) on the neck and shoulder region, but had a long-standing spinal disc herniation detected by MRI of the spine. In patients 2 and 3, the symptomatology almost disappeared in each painful area (VAS 1). In patient 4, there was only residual symptomatology on the TMJ (VAS 4). In patient 5, the symptomatology completely disappeared in each painful area (VAS 0). There was a significant effect for VAS [t(4) = 7.9, *p* = 0.0013] (**Table 4**).

Each of the patients was fully compliant with the “prescribed” home treatment program.

Tables 2.1, 2.2 summarize the impact of the gnathological treatment concerning trigger points (palpation). Compared with baseline (T1), posttreatment (T2) pain extent and intensity during palpation of the masseter, temporal, sternocleidomastoid, digastric, and pterygoid decreased in all patients.

**Table 2.1:** Masseter, temporal, and sternocleidomastoid palpation test after treatment compared with baseline.

	**Masseter palpation at T1**	**Masseter palpation at T2**	**Temporal palpation at T1**	**Temporal palpation at T2**	**Sternocleidomastoid palpation at T1**	**Sternocleidomastoid palpation at T2**
PT 1 (CS)	3	1	2	1	3	1
PT 2 (SS)	3	1	3	1	3	0
PT 3 (RF)	2	0	2	0	3	1
PT 4 (AN)	3	2	2	0	3	1
PT 5 (CT)	2	0	2	1	2	0

**Table 2.2:** Digastric and pterygoid palpation test after treatment compared with baseline.

	**Digastric palpation at T1**	**Digastric palpation at T2**	**Pterygoid palpation at T1**	**Pterygoid palpation at T2**
PT 1 (CS)	1	0	3	1
PT 2 (SS)	2	0	3	0
PT 3 (RF)	2	0	3	1
PT 4 (AN)	0	0	3	2
PT 5 (CT)	1	0	2	0

According to the VAS data for patients 2, 3, and 5, the pain evoked by masseter, temporal, and sternocleidomastoid muscle palpation almost disappeared (PALPATION TEST 0–1). Patient 1 exhibited the presence of spinal disc herniation; it caused no persistency in terms of pain upon palpation of the masseter, temporal, and sternocleidomastoid muscles (PALPATION TEST 1). Patient 4 exhibited persistent pain at the masseter muscle upon palpation (PALPATION TEST 2) due to the residual pain experienced in the TMJ.

Pain on palpation in the digastric muscle region disappeared in all patients (PALPATION TEST 0). Pain on palpation in the pterygoid muscle region was relevant only in patient 4 (PALPATION TEST 2) with residual TMJ pain. There was a significant effect for palpation of the masseter [t(4) = 9, *p* < 0.001], temporalis [t(4) = 6.5, *p* = 0.0028], sternocleidomastoid [t(4) = 11, *p* < 0.001], digastric [t(4) = 3.2, *p* = 0.0326], and pterygoid [t(4) = 6.3, *p* = 0.0032] (**Table 4**).

**Figure 2:**
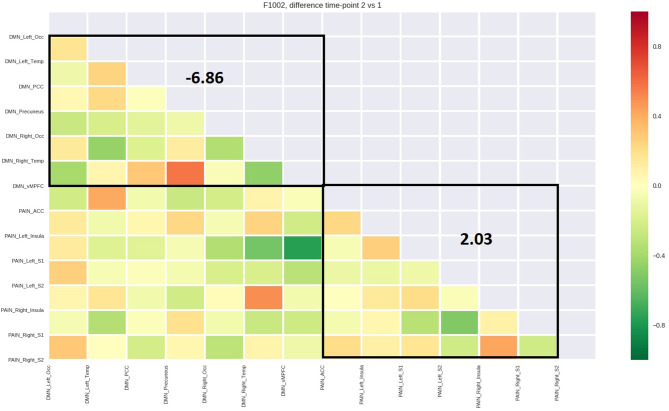
Matrix difference T2–T1 of the first patient and DMN and PN average connectivity.

**Figure 3:**
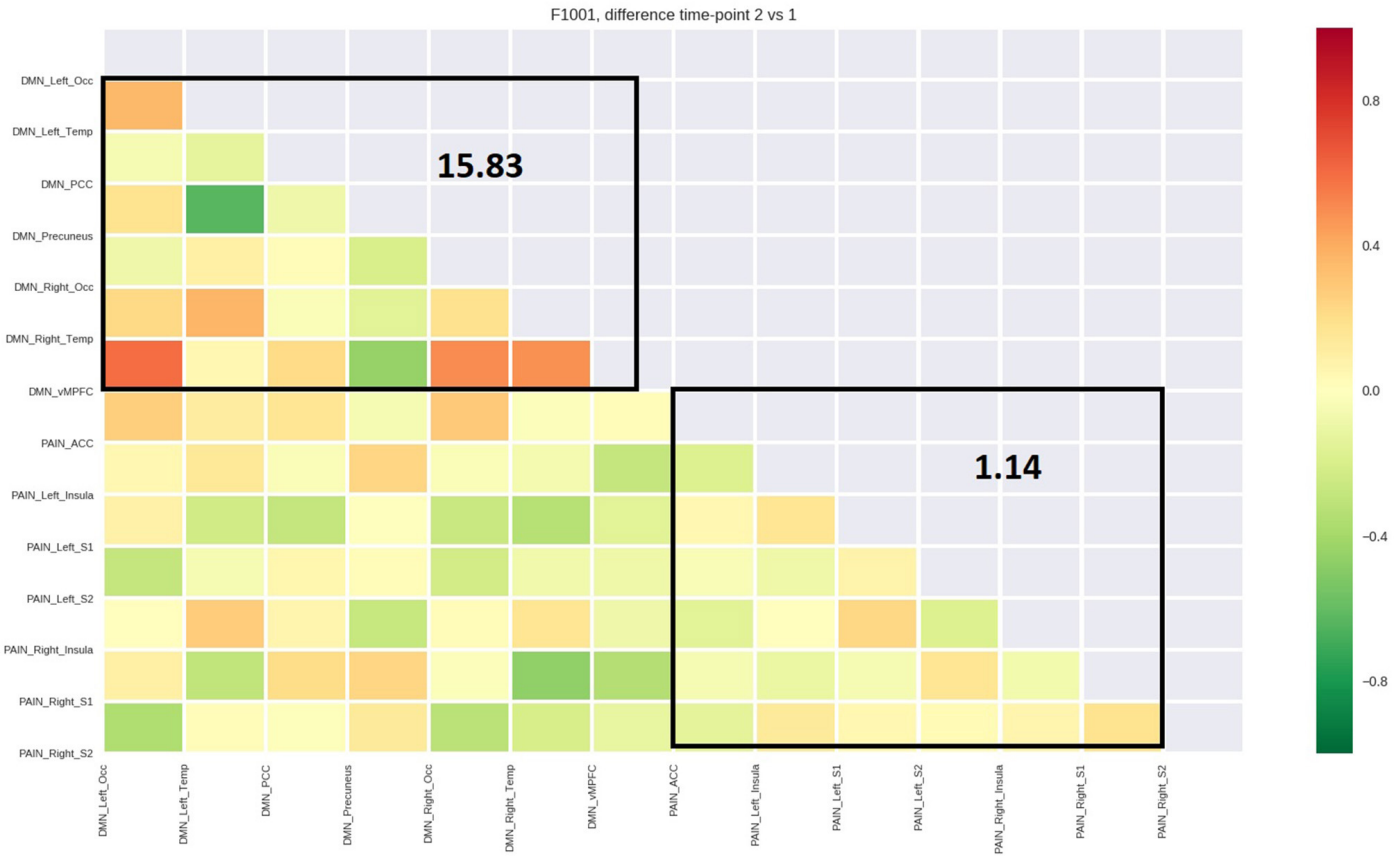
Matrix difference T2–T1 of the second patient and DMN and PN average connectivity.

**Figure 4:**
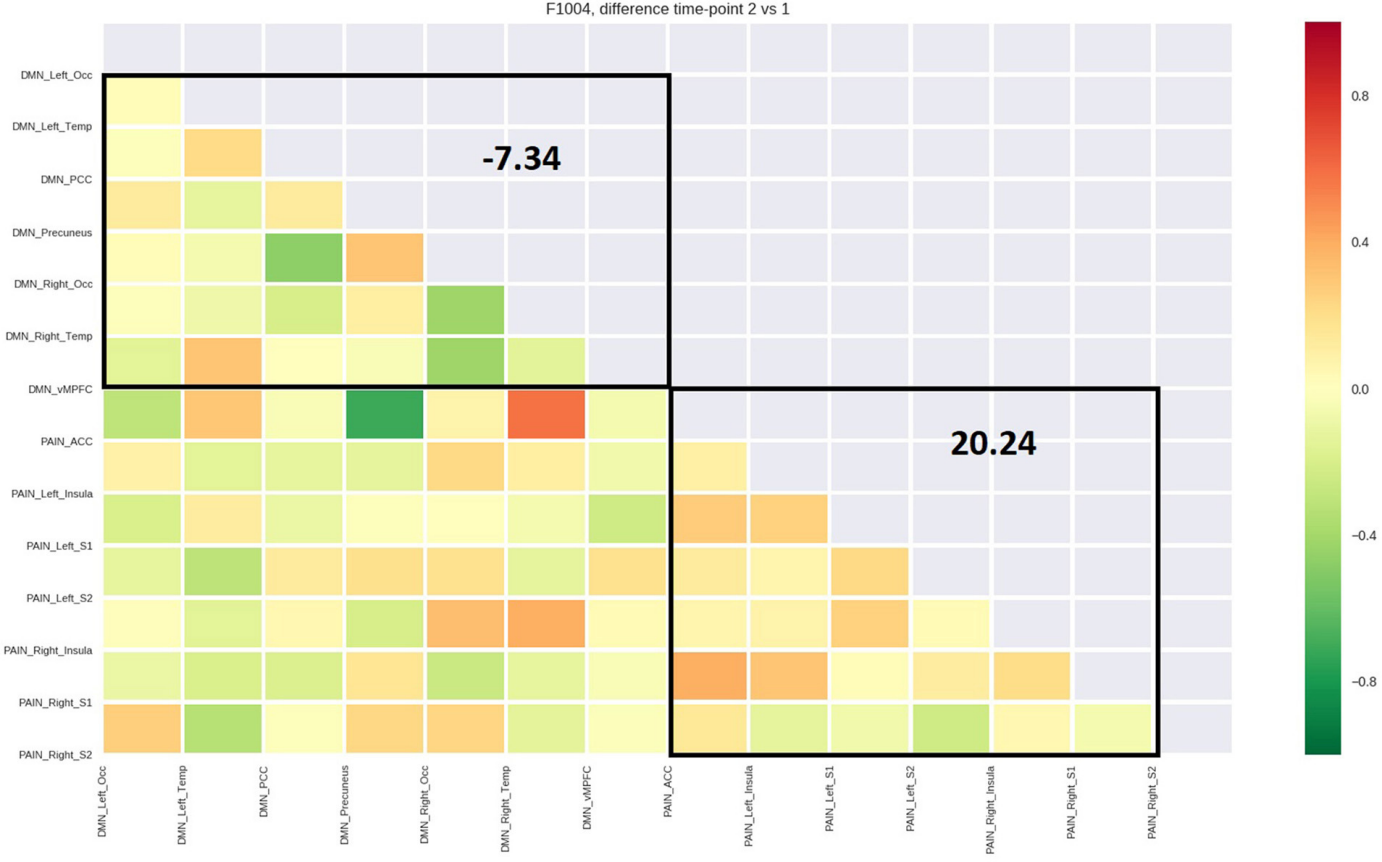
Matrix difference T2–T1 of the third patient and DMN and PN average connectivity.

**Figure 5:**
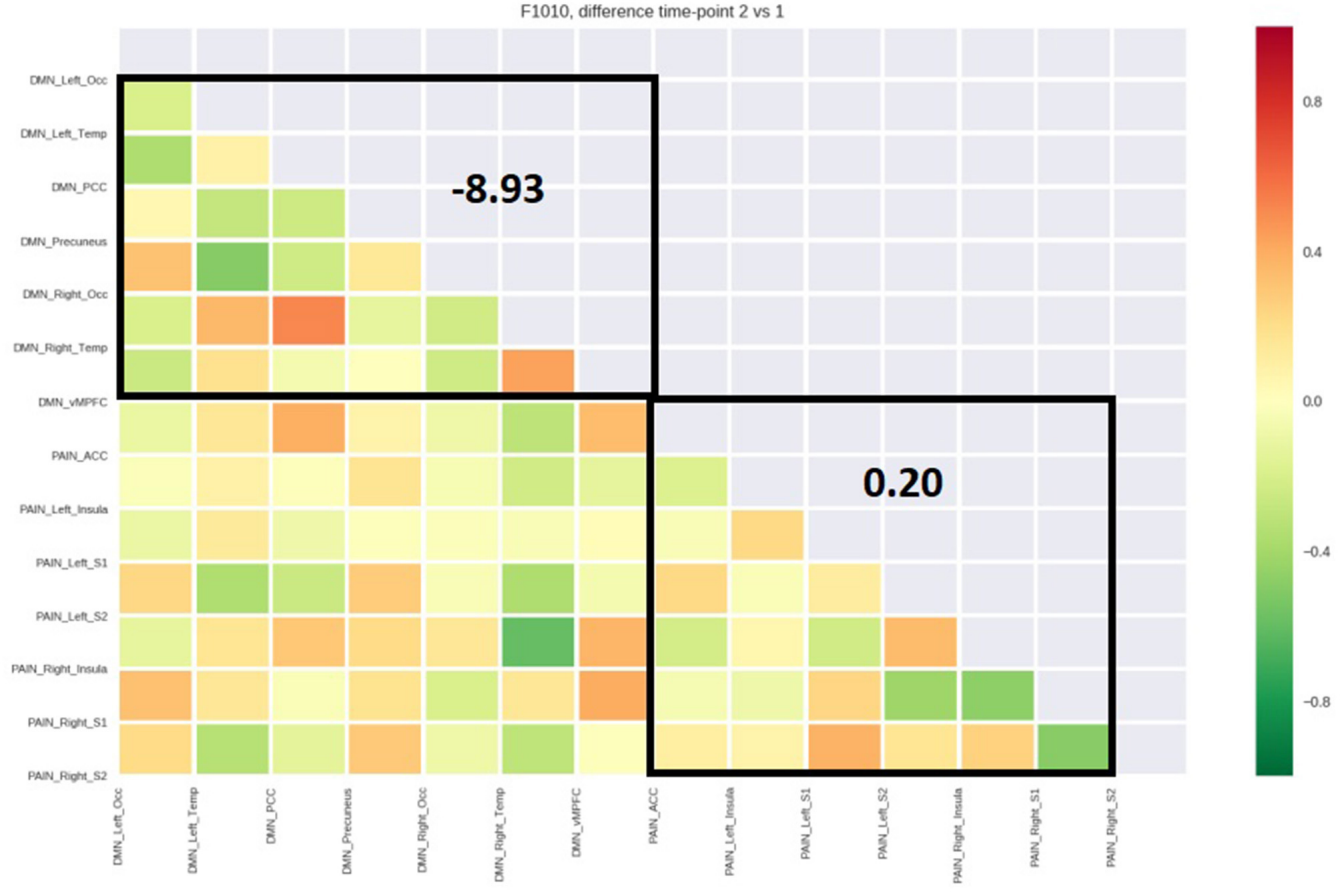
Matrix difference T2–T1 of the fourth patient and DMN and PN average connectivity.

**Figure 6:**
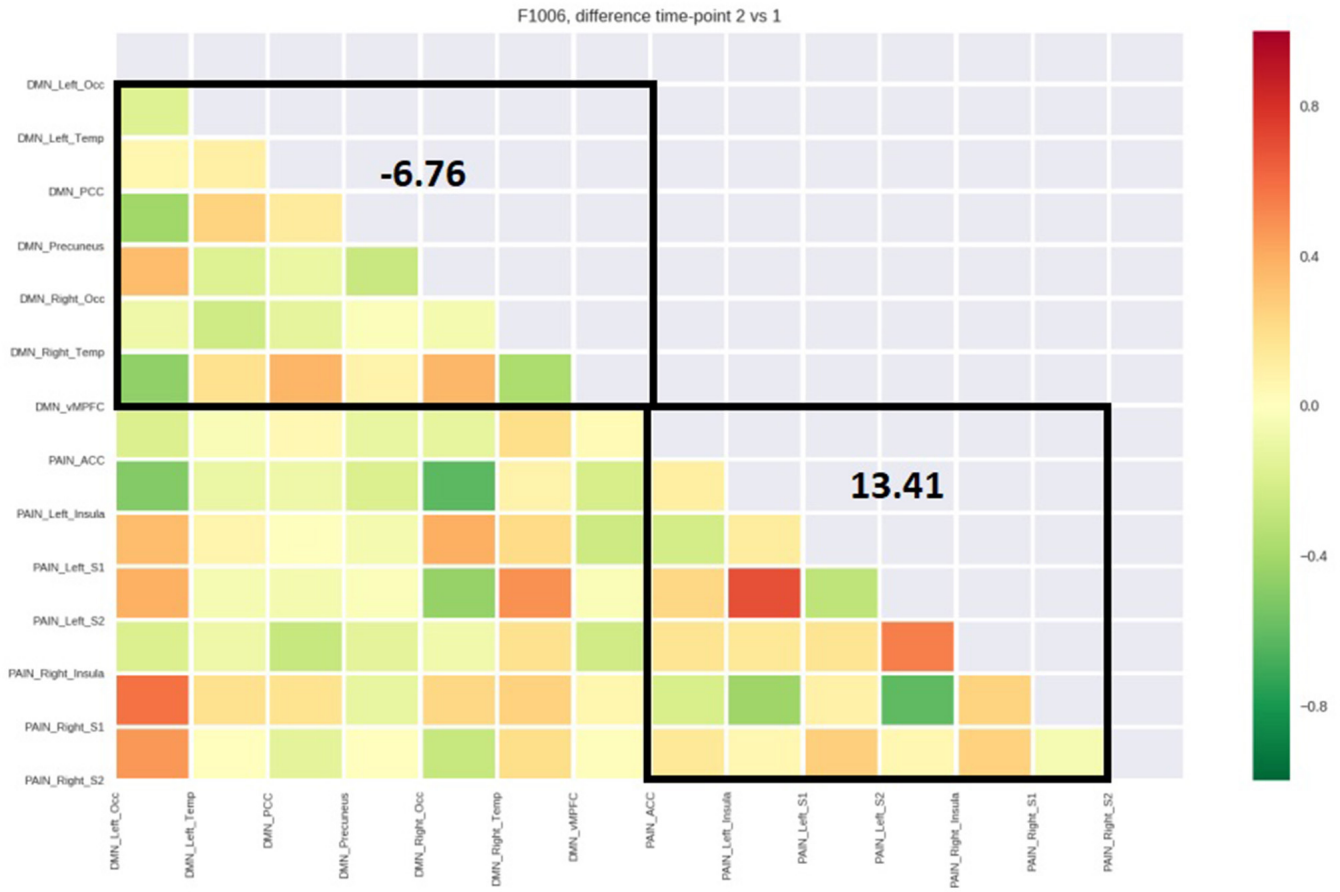
Matrix difference T2–T1 of the fifth patient and DMN and PN average connectivity.

Table 3 reports the average functional connectivity, which in the PN had a positive value for all five patients while in the DMN had a negative value for four of five patients. The variations of fcMRI within each network were uniform among the five patients. Only patient 1's DMN behaved differently, possibly due to the detected spinal disc herniation. Figures 2–6 show the matrix difference T2–T1 for all ROIs of PN and DMN for each patient; the algebraic sum of the functional connectivity values of each ROI in this matrix was represented as the average connectivity of each network. There was no significant effect for the DMN average connectivity [t(4) = −0.6, *p* = 0.5801] and for the PN average connectivity [t(4) = 1.8, *p* = 0.1382] (Table 4).

**Table 3:** Average connectivity of the DMN and PN.

	**DMN average**	**PN average**
	**connectivity: T2–T1**	**connectivity: T2–T1**
PT 1 (CS)	15.83	1.14
PT 2 (SS)	6.86	2.03
PT 3 (RF)	7.34	20.24
PT 4 (AN)	6.76	13.41
PT 5 (CT)	8.93	0.20

**Table 4:** Statistical results.

	**Masseter**	**Temporal**	**Sternocleidomastoid**	**Digastric**	**Pterygoid palpation**	**VAS**	**DMN**	**PN**
	**palpation**	**palpation**	**palpation**	**palpation**	**at T1**			
t.stat (paired)	9	6.531972647	11	3.207134903	6.32455532	7.90569415	−0.601279077	1.848546182
df	4	4	4	4	4	4	4	4
t-crit	2.776445105	2.776445105	2.776445105	2.776445105	2.776445105	2.776445105	2.776445105	2.776445105
*p*	<0.001	<0.1	<0.001	<0.05	<0.01	<0.01	>0.05	<0.05
sig	sig	sig	sig	sig	sig	sig	no. sig	no. sig

## Discussion

In this case series of five patients with myofascial pain in the masticatory region due to unconscious clenching reflexes, we investigated the impact of gnathological therapies on pain and associated arrangement of pain and default mode neural networks. Teeth clenching is an unconscious behavior that represents the etiology of all TMDs, which have complex and multifactorial etiology outcomes. A scientific review reveals the TMDs' major causal factors including occlusion, trauma, emotional stress, deep nociceptive stimuli, and parafunctional activities (bruxism and clenching). If there are substantial loads (e.g., unconscious clenching), slight flexion in the mandible causes tension in the discal ligaments and intracapsular disorders. The unconscious clenching and bruxism cause microtrauma against the teeth, muscles, and joints.

The most frequent TMD associated with unconscious clenching is myofascial pain syndrome, the fourth stage of muscular pathology in TMD (16). Tooth clenching diagnosis is made when the patient reports problems such as tension headaches, neck pain, back pain, tenderness of the masticatory muscles, and fatigue of the masticatory muscles when chewing hard food, difficulty opening the mouth completely, muscle tenderness on waking, and muscular tension in the head–neck region. Myofascial pain is different compared with migraine pain because headaches respond to anti-inflammatories and are independent of noises and lights. Also, myofascial pain can have bilateral involvement, and it is not excessively debilitating.

Biofeedback has been used for over 50 years in muscle rehabilitation to facilitate standard movement patterns after injury. This technique provides biological information, which would not otherwise be known by the patient in real-time (17). A systematic review of the literature concludes that biofeedback can be useful in helping to manage masticatory muscle activity. Most of the studies showed a significant correlation between the use of biofeedback and the reduction of masticatory muscle activity (18).

The study outlined a significant decrease in the symptomatology (Tables 1, 2.1, 2.2, 4), both in terms of the referred pain (VAS) and in terms of trigger points detected (assessed by muscle palpation). The TMJ MRI did not highlight any modification in the disc–condyle relation in patients diagnosed with either combined intra- and extra-articular disorder or only extra-articular disorder. However, the treatment was equally effective for both subgroups of patients in terms of reduction of pain, unconscious teeth clenching, and muscular tension. fMRI of the brain revealed that in all the patients, the average fcMRI of the pain network tended to increase, whereas after the treatment, the average fcMRI of the DMN tended to decrease in four out of five patients (Tables 3, 4, and Figures 2–6). It is proposed to increase the sample size to permit exploration of the data using robust statistical methods.

Our finding of lower functional connectivity of the DMN, associated with a greater functional connectivity of the PN after the treatment, is consistent with the results of a recent study showing that DMN and the PN are functionally connected but show an inverse temporal modulation (19). It is important to emphasize that a decrease or increase in functional connectivity at rest within a neural network does not correspond to an increase or decrease in the physiological activity of that network; it may instead indicate an increase or decrease of the task-evoked activity.

This study research outlines limits concerning low sample sizes; therefore, the aim for the future is to increase the sample size; furthermore, another limit is the no use of EMG, on which inclusion in future studies will be relevant. Based on our qualitative and quantitative clinical and fcMRI evaluation of five patients, our data suggest that a gnathological treatment protocol based on a splint and associated biofeedback exercises is effective and repeatable in future studies. Moreover, we showed the neurophysiological impact of the gnathological therapy, assessed as qualitative clinical/quantitative fMRI PN and DMN measurements of improvement. After the treatment was completed, the functional connectivity of the brain networks showed homogeneous changes in all the five patients.

## Data Availability Statement

The original contributions presented in the study are included in the article/supplementary material, further inquiries can be directed to the corresponding author/s.

## Ethics Statement

The studies involving human participants were reviewed and approved by University of Chieti Ethics Committee. The patients/participants provided their written informed consent to participate in this study.

## Author Contributions

MM and CR submitted the study protocol to the ethics committee and wrote the manuscript. FF, MM, and CR selected the sample. MC and RN performed the fMRI and analyzed the radiological data. FF, MM, AS, and CR analyzed the clinical data. All authors read and approved the final manuscript.

## Conflict of Interest

The authors declare that the research was conducted in the absence of any commercial or financial relationships that could be construed as a potential conflict of interest.

## Abbreviations

TMD: temporomandibular disorder
TMJ: temporomandibular joint
fMRI: functional magnetic resonance imaging
BOLD: blood oxygenation level dependent
fcMRI: functional connectivity magnetic resonance imaging
MRI: magnetic resonance imaging
PN: pain network
DMN: default mode network
LPAS: lower passive aligner splint
UPAS: upper passive aligner splint
T1: time one (is for time-points)
T2: time two (is for time-points).

## References

[1] Brett K Wells C Sinclair A Tenenbaum H Freeman B Spry C. Interventions for Temporomandibular Joint Disorder: An Overview of Systematic Reviews. Ottawa, ON: Canadian Agency for Drugs and Technologies in Health. (2018). 31361423

[2] Andreescu C Aizenstein H. Predicting treatment response with functional magnetic resonance imaging. Biol Psychiatry. (2016) 79:262–3. 10.1016/j.biopsych.2015.11.017 26796875

[3] Oda M Yoshino K Tanaka T Shiiba S Makihara E Miyamoto I. Identification and adjustment of experimental occlusal interference using functional magnetic resonance imaging. BMC Oral Health. (2014) 14:124. 10.1186/1472-6831-14-124 25304016 PMC4200220

[4] Raichle ME Mintun MA. Brain work and brain imaging. Annu Rev Neurosci. (2006) 29:449–76. 10.1146/annurev.neuro.29.051605.112819 16776593

[5] Nishiyama A Otomo N Tsukagoshi K Tobe S Kino K. The true-positive rate of a screening questionnaire for temporomandibular disorders. Open Dent J. (2014) 8:236–40. 10.2174/1874210601408010236 25614769 PMC4298036

[6] Nixdorf DR John MT Wall MM Fricton JR Schiffman EL. Psychometric properties of the modified Symptom Severity Index (SSI). J Oral Rehabil. (2010) 37:11–20. 10.1111/j.1365-2842.2009.02017-x 19889036 PMC2858780

[7] Fischl B Salat DH Busa E Albert M Dieterich M Haselgrove C. Whole brain segmentation: automated labeling of neuroanatomical structures in the human brain. Neuron. (2002) 33:341–55. 10.1016/s0896-6273(02)00569-x 11832223

[8] Mennes M Biswal BB Castellanos FX Milham MP. Making data sharing work: the FCP/INDI experience. Neuroimage. (2013) 82:683–91. 10.1016/j.neuroimage.2012.10.064 23123682 PMC3959872

[9] Brett M Johnsrude IS Owen AM. The problem of functional localization in the human brain. Nat Rev Neurosci. (2002) 3:243–9. 10.1038/nrn756 11994756

[10] Jenkinson M Beckmann CF Behrens TE Woolrich MW Smith SM. FSL. Neuroimage. (2012) 62:782–90. 10.1016/j.neuroimage.2011.09.015 21979382

[11] Nebel MB Folger S Tommerdahl M Hollins M McGlone F Essick G. Temporomandibular disorder modifies cortical response to tactile stimulation. J Pain. (2010) 11:1083–94. 10.1016/j.jpain.2010.02.021 20462805 PMC2943972

[12] van Rossum G. Python tutorial, Technical Report CS-R9526, Centrum voor Wiskunde en Informatica (CWI). Amsterdam (1995).

[13] Lundh H Westesson PL Eriksson L Brooks SL. Temporomandibular joint disk displacement without reduction. Treatment with flat occlusal splint versus no treatment. Oral Surg Oral Med Oral Pathol. (1992) 73:655–8. 10.1016/0030-4220(92)90003-9 1437030

[14] Dworkin SF LeResche L. Research diagnostic criteria for temporomandibular disorders: review, criteria, examinations and specifications, critique. J Craniomandib Disord. (1992) 6:301-55 1298767

[15] Headache Classification Committee of the International Headache Society (IHS). The International Classification of Headache Disorders, 3rd edition (beta version). Cephalalgia. (2013) 33:629–808. 10.1177/0333102413485658 23771276

[16] Okeson JP. Management of Temporomandibular Disorders and Occlusion-E-Book. Elsevier Health Sciences (2019).

[17] Giggins OM Persson UM Caulfield B. Biofeedback in rehabilitation. J Neuroeng Rehabil. (2013) 10:60. 10.1186/1743-0003-10-60 23777436 PMC3687555

[18] Florjanski W Malysa A Orzeszek S Smardz J Olchowy A Paradowska-Stolarz A. Evaluation of biofeedback usefulness in masticatory muscle activity management—a systematic review. J Clin Med. (2019) 8:766. 10.3390/jcm8060766 31151198 PMC6616888

[19] Mantini D Caulo M Ferretti A Romani GL Tartaro A. Noxious somatosensory stimulation affects the default mode of brain function: evidence from functional MR imaging. Radiology. (2009) 253:797–804. 10.1148/radiol.2533090602 19789220

